# Peer relationships, adolescent anxiety, and life satisfaction: a moderated mediation model in Turkish and syrian samples

**DOI:** 10.1007/s00787-023-02366-7

**Published:** 2024-02-01

**Authors:** Onat Yetim, Resul Çakır, Ece Bülbül, İlham Sebea Alleil

**Affiliations:** 1 Psychology Department, Toros University, Bahçelievler District, Mersin, Turkey; 2 Mersin, Turkey

**Keywords:** Adolescent, Peer relationship, Syrian refugee, Satisfaction with life

## Abstract

Prior studies comparing Syrian refugee adolescents to their native peers in the same region have found higher anxiety and lower life satisfaction. Therefore, identifying regulatory variables is crucial for implementing support programs. This study examined the mediating effect of peer relationships and the moderating effect of being a refugee or native adolescent on the relationship between adolescent anxiety and life satisfaction across different samples. Participants and setting: The study included 2,336 adolescents aged 11–19 (M = 14.79, SD = 1.04). Participants completed the Screen for Child Anxiety Related Disorders, Satisfaction with Life Scale, and Strengths and Difficulties Questionnaire. The mediation and moderation effects were analyzed with the path analysis codes written on Mplus 8.3. SPSS 26 was used for descriptive statistics and group comparisons. The findings showed that peer relationships mediate adolescent anxiety and life satisfaction, and this relationship is moderated according to whether the participants are native adolescents or refugee adolescents. This study highlights the significant associations between peer relationships, adolescent anxiety, and life satisfaction and the moderating role of the participant identity. The findings may inform psychological interventions to improve Syrian refugee adolescents' mental health and well-being. These findings may also have implications for policies and programs aimed at supporting the integration of Syrian refugee adolescents in host communities.

## Introduction

As a result of the ongoing civil war in Syria for over a decade, millions of Syrians have been forced to leave their homes [131]. Turkey has emerged as the most important destination for Syrians who have been forced to flee their homeland following the war [131]. In this context, Turkey is hosting 3,648,983 Syrian refugees [49]. Additionally, many Syrian refugees worldwide and in Turkey consist of children and adolescents [17, 43, 49]. Although settling in a different country affects the entire family, children and adolescents tend to attach more emotions and significance to this experience than adults, and they are more greatly impacted by it [5, 14, 83].

### Anxiety and satisfaction with life among refugee adolescents

Adolescence is a crucial stage marked by significant cognitive and physical changes. The challenges inherent in this period are further intensified when adolescents undergo stressful life events, such as relocating to a new country or experiencing the impact of war [41, 47]. These adolescents have been exposed to traumatic experiences, challenging living conditions, separation from family members, and the loss of loved ones [86]. A study conducted by Özer et al. [97] on refugee adolescents in Turkey reported that adolescents between the ages of 9 and 18 had experienced numerous traumatic events, with 74% witnessing the loss of a loved one. Adolescents who are in a critical period of their lives after resettlement are particularly at higher risk and face significant problems [10, 71].

In addition to the stressful experiences they faced in their own countries and the everyday stress of adolescence, factors such as family traumas in the host country, differences in the education system, and language barriers contribute to significant challenges and increased vulnerability for refugees after resettlement [13, 48, 69]. Bean et al. [10] and Khamis [69] state in their studies on refugee adolescents that a significant relationship exists between stressful life events and psychiatric disorders. Anxiety is one of the most common psychiatric disorders observed in these adolescents [21, 55, 57, 58, 135]. Many researchers in the literature, both in Turkey and in different countries, have conducted studies on Syrian refugee adolescents and have noted that at least half of these adolescents exhibit symptoms of anxiety [25, 52, 64, 66]. Similarly, studies comparing refugee adolescent samples with local samples have found that refugee adolescents have higher rates of anxiety compared to the local sample [18, 45, 88, 110, 134, 146].

Children and adolescents affected by war not only experience trauma and related psychopathologies but also exhibit various emotional and behavioral problems. These problems include self-harm, involvement in criminal activities, hyperarousal, decreased life satisfaction, and decreased social functioning [6, 13]. Indeed, in a study conducted by Al-Masri et al. [3] comparing Syrian immigrants with the local population in Germany, it was found that Syrian immigrants had lower life satisfaction than the local population.

### The influence of post-migration social relationships on anxiety and satisfaction with life

Linking the traumatic experiences of refugee adolescents solely to pre-migration stress factors leaves out many other significant issues [13]. Therefore, studies conducted after resettlement become crucial. After settling in the host country, refugee adolescents are exposed to negative experiences such as language problems, difficulties in adapting to the new environment, and discrimination [80]. Furthermore, there are studies indicating that refugee adolescents struggle to adapt to the school environment, experience peer bullying, and encounter peer-related problems [31, 44, 108, 145].

Adolescents are influenced by how their peers perceive them [92]. Moreover, negative experiences in their social relationships can have psychiatric consequences for adolescents [95]. Many researchers in the literature state that peer interactions impact adolescents' development and well-being [27, 105]. For example, it is known that there is a negative relationship between social support and anxiety during adolescence [87, 114, 115]. For instance, a study conducted by Havewala et al. [56] suggests that receiving support from peers and classmates can decrease adolescent anxiety levels. Al-Shatanawi et al. [4] state that social isolation and loneliness are among the observed primary psychiatric disorders in Syrian refugee adolescents. Therefore, peer relationships are highly important for refugee adolescents and play a critical role in reducing the psychiatric symptoms observed in this sample [15, 19, 69].

The devastating effects of traumatic experiences and exposure to war cannot be solely alleviated through positive relationships with peers. However, refugee adolescents, especially those who have to resettle in a different country and spend more time with their peers, are more frequently exposed to stress factors originating from their peers [106]. Establishing positive relationships with local peers after resettlement helps reduce adjustment difficulties and increase life satisfaction for these refugee adolescents [31, 38, 46, 81, 99, 112, 130, 133].

It is well-known that adolescence is a period characterized by uncertainties, and it is the time when anxiety disorders are most commonly observed [26, 39, 84, 101, 104, 118–120, 139, 141]. Therefore, anxiety is an important variable for native Turkish adolescents as well. Anxiety in Turkish adolescents is considered one of the main factors negatively impacting their life satisfaction [67].

In light of these findings, it is expected that peer relationships play a mediating role in the relationship between anxiety and life satisfaction. Thus, uncovering the role of peer relationships in the anxiety-life satisfaction relationship for both native and Syrian refugee adolescents is a crucial aspect that needs to be explored.

### The current study

Since the beginning of the war in Syria, many researchers have conducted studies on Syrian refugees and refugee adolescents (e.g., [12, 20, 79, 137]). When examining the limited number of studies conducted on refugee adolescents, it can be observed that studies with Syrian adolescents mainly focus on pre-migration risk factors. Although some studies have explored factors such as family [41] and well-being [103] after the resettlement of refugee adolescents in the host country, peer relationships have been neglected in this sample [32]. In this context, it is evident that sufficient attention has not been given to protective factors for the mental health of Syrian refugee adolescents.

Moving to a new country is a stressful life event that causes significant changes in one's life and can have lasting negative effects from childhood to adulthood, making refugees vulnerable to mental health problems [62]. It is known that 70% of adult mental health problems begin in adolescence [33]. The high prevalence of anxiety among Syrian refugee adolescents compared to the general population, the strong predictor of potential negative outcomes in adulthood associated with adolescent anxiety [100], and its co-occurrence with various psychiatric disorders [42], low life satisfaction [8, 82], and low academic performance [70, 121], highlight the importance of interventions during this period.

Based on these findings, it is important to identify potential risk and social factors for anxiety disorders, low life satisfaction, and social adaptation among refugee adolescents. Although there have been numerous studies on refugee adolescents in the literature, a limited number of studies examine the impact of peer relationships. The present study aims to investigate the mediating effect of peer relationships on anxiety levels and life satisfaction and to explore the potential moderating effect of being a refugee adolescent or a local Turkish adolescent on this mediation mechanism in Turkey.

### Research hypotheses

**Fig. 1:**
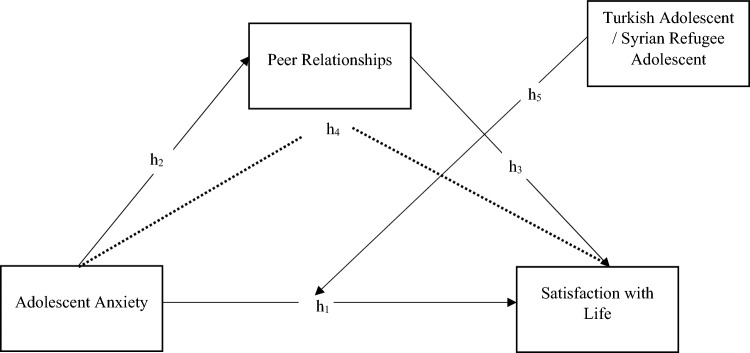
Research Model

The present study assumes that (a) there will be a negative relationship between adolescent anxiety and life satisfaction, (b) adolescent anxiety will be negatively associated with peer relationships, (c) peer relationships will be positively related to life satisfaction, (d) peer relationships will mediate the relationship between adolescent anxiety and life satisfaction, and (e) being a local Turkish adolescent or a Syrian refugee adolescent will have a moderating effect on this mediation relationship (See Fig. 1).

## Method

### Participants

This study’s Sample included 2,336 individuals aged between 14 and 19 years Turkish Sample: M = 15.02, SD = 0.86; Syrian Sample: M = 14.56, SD = 1.15). Data for the study were collected from two distinct groups comprising 1148 Syrian adolescent refugees (Sample 2) who migrated to Turkey due to the war and 1188 Turkish students (Sample 1). Of the total participants, 1532 were female, and 804 were male. The data collection process was conducted across four different cities in Turkey, namely Aksaray, Hatay, Kahramanmaraş, and Mersin.

### Procedure

An exploratory quantitative research design guided the cross-sectional sampling method to recruit adolescents from diverse schools and non-governmental organizations in Turkey. The selection of the research design was based on the strengths of the established quantitative methods, allowing for flexible adoption of the method. Relying on convenience sampling, public schools and non-governmental organizations in Turkey were approached and invited to participate in the research. Written informed consent was obtained from a parent or legal guardian, followed by written assent of the participant if they were younger than 18. Written consent was obtained from the participants at least 18 years of age. Students who completed the consent form knew how to read and write in their native language (Turkish or Arabic) and volunteered to participate in the study were included in the data collection process. As a result, the participation rate in the research was calculated as 89%. Considering the recommendations of Bryman and Cramer [23] and Tabachnick and Fidel [123] to calculate the sample size according to the number of scale items, 2568 students who agreed to participate in the study were included. After invalid data was checked, the number of participants decreased to 2,336.

During the data collection process, either one of the researchers or the psychological counselor appointed by the relevant educational institution supervised the students’ possible problems by addressing their queries and concerns. During this process, the researcher or the psychological counselor provided no guidance, and the students freely responded. Furthermore, it is noteworthy that the data collection process for Syrian adolescents was facilitated by a researcher fluent in Arabic, their native language. Turkish and Arabic scales were applied to the participants in the data collection process according to their native languages. The present study complied with the regulations stipulated by the University Ethics Committee, and data collection was performed between January and April 2023.

### Measurements

Screen for Child Anxiety Related Disorders (SCARED) was used in the current study to assess anxiety symptoms. The SCARED was developed by Birmaher et al. [16] to evaluate anxiety disorder symptoms in children and adolescents as well as for screening purposes. The SCARED scale consists of 41 items (e.g., I worry about how well I'm doing things) that are scored on a 3-point Likert scale (0 = Not true, 2 = Very true or often true) and includes five subscales measuring panic disorder, somatic disorder, generalized anxiety disorder, separation anxiety, and social anxiety. The total score ranges from 0 to 82, with higher scores indicating higher levels of the corresponding trait. However, a score of 25 or higher on the SCARED indicates a warning for anxiety disorders.

The scale was adapted to Turkish culture by Cakmakci [24]. In this study, Cronbach's alpha internal consistency coefficient of the scale was reported to be between 0.74 and.93.The scale adapted to Arabic culture by Hariz et al. [54]. In this study, Cronbach's alpha internal consistency coefficient of the scale was reported to be between 0.65 and 0.89. In Birmaher et al.'s [16] study, Cronbach's alpha reliability coefficient for the scale and subscales ranged from 0.74 to 0.93, and the test–retest reliability coefficient ranged from 0.70 to 0.90. In the current study, Cronbach's alpha reliability coefficient for SCARED was calculated as 0.90 for the Turkish sample, 0.85 for the Syrian sample and 0.92 for the whole.

Satisfaction with Life Scale (SWLS) was used to assess participants' life satisfaction. The SWLS was developed by Diener et al. [35] to evaluate an individual's subjective evaluation of their overall life satisfaction. The scale consists of 5 items (e.g. I have a life close to my ideals in many ways) and a single subscale evaluated on a 7-point Likert scale (1 = Not at all appropriate, 7 = Very appropriate). An overall score on the SWLS ranges from 5 to 35, with higher scores indicating greater life satisfaction. Köker [74] adapted the scale to Turkish culture. In this study, the test–retest reliability coefficient was 0.85. Yetim [144] reported a Cronbach's alpha reliability coefficient of 0.86 and a test–retest reliability coefficient of 0.75. Abdallah [1] adapted the scale to Arabic culture. In this study, Cronbach's alpha internal consistency coefficient for the scale's total score was calculated as 0.79. In the current study, Cronbach's alpha internal consistency coefficient was calculated as 0.75 for the Turkish sample and 0.70 for the Syrian sample.

Strengths and Difficulties Questionnaire (SDQ) originally developed by Goodman [51], was used to measure peer problems. The SDQ is a widely used tool to assess emotional and behavioral problems in children aged 4–16. The SDQ contains 25 items, each scored on a 3-point Likert scale (0 = Not True, 1 = Somewhat True, 2 = Certainly True), and is composed of five subscales: hyperactivity/inattention, behavioral problems, emotional problems, peer problems, and prosocial behavior. The peer problems subscale, which consists of five items, was selected for the current study. The total score that can be obtained from the subscale varies between 0 and 10, and an increase in the score indicates a positive peer relationship.

As proposed for adolescents, some items in this subscale appear to reflect loneliness (e.g., feeling rather solitary, tending to be alone, having at least one good friend) and sociability (e.g., generally liked by other people my age; getting on better with adults than with people my own age) [140]. Additionally, the peer problems subscale has been shown to moderately correlate with adolescents' internalizing symptoms [96]. Therefore, we chose this for the current study.

Güvenir et al. [53] adapted the scale to Turkish culture. In this study, Cronbach's alpha internal consistency coefficient for the peer problems subscale was 0.37. The scale was adapted to the Arabic adolescent sample by Mukhaini et al. [90] and Emam et al. [40]. In these studies, Cronbach's alpha internal consistency coefficient for peer problems subscale was reported as 0.30 and 0.40, respectively. The present study calculated Cronbach's alpha internal consistency coefficient as 0.70 and 0.40 for the Turkish and Syrian samples, respectively.

### Statistical approach

The moderated mediating effect occurs when the relationship between the independent and dependent variables varies at different levels of the moderator variable [91]. With this perspective in our study, we tested the second-stage moderated mediation effect, which means the moderator (e.g., participant sample) moderated the association between the mediator (e.g., peer relationship) and satisfaction with life.

In this study, we employed SPSS 26.0 and Mplus 8.3 [93] to conduct descriptive statistics and moderated mediation analyses. A pre-determined alpha level of 0.05 was used to determine the statistical significance of all analyses conducted in this study. Before the analysis, the normality assumptions of the data were tested using the skewness and kurtosis coefficients. As a result of this analysis, it was determined that all research variables were within acceptable limits [50], maximum skewness = 1.191, maximum kurtosis = 1.489). We conducted descriptive statistics and correlation analysis using SPSS 26.0 first. Subsequently, we employed Mplus 8.3 for the analysis of moderated mediation. When the moderated mediating effect was significant, we followed Cohen et al.'s [29] recommendations and plotted the two slopes, interpreting the nature of these multiple models (Table 1).

**Table 1:** Distribution of demographic characteristics

Variables	Groups	n	%
Turkish sample			
1. Gender	Female	674	56.8
	Male	514	43.3
2. Attendance status	Attending	1188	100
	Not attending	0	0
3. Employment status	Employed	42	3.5
	Unemployed	1146	96.5
Syrian sample			
1. Gender	Female	858	74.7
	Male	290	25.3
2. Attendance status	Attending	1004	87.5
	Not attending	136	11.8
3. Employment status	Employed	168	14.6
	Unemployed	947	84.8
4. Turkish proficiency	Low proficiency	93	8.1
	Moderate proficiency	514	44.8
	High proficiency	538	46.9

## Results

### Preliminary analyses

The means, standard deviations, skewness, kurtosis, and Cronbach alpha internal consistency coefficients among the variables are shown in Table 2 according to the sample of the participants.

**Table 2:** Descriptive, scale reliability, and inferential statistics

Variables	Means	Standard deviations	Cronbach Alpha	Skewness	Kurtosis
Turkish sample					
1. SCARED	32.58	13.81	0.97	0.430	− 0.113
2. SWL	20.12	6.16	0.75	− 0.718	0.833
3. PR	4.04	2.92	0.70	0.510	− 0.174
Syrian sample					
1. SCARED	56.28	13.59	0.84	1.191	1.489
2. SWL	15.20	6.89	0.70	− 0.290	0.025
3. PR	2.52	3.01	0.40	− 0.104	0.052
Total					
1. SCARED	44.23	18.11	0.92	0.028	− 0.679
2. SWL	17.70	6.98	0.91	− 0.171	− 0.595
3. PR	3.29	3.06	0.62	0.858	− 0.928

*SCARED* screen for child anxiety-related disorders, *SWL* satisfaction with life, *PR* peer relationship

The means, standard deviations, and point biserial correlations among the variables are shown in Table 3. Point biserial correlation is the value of the Pearson product correlation when one of the variables is dichotomous and the other is metric [73, 113]. As we expected, adolescent anxiety is negatively correlated with life satisfaction (*r* = − 0.26, *p* < 0.01) and peer relationships (*r* = − 0.10, *p* < 0.01). In contrast, peer relationships are positively correlated with life satisfaction (*r* = 0.18, *p* < 0.01). ANOVA analysis was conducted to examine whether the anxiety and life satisfaction rates of the participants changed according to the local or refugee sample (see Table 4). According to the results of the analysis, it was observed that the adolescent anxiety [*f*(1,2335) = 1745,852, *p* = 0.000] levels of the Syrian refugee adolescents were higher than the local sample, and their life satisfaction was lower [*f*(1,2335) = 331,593, *p* = 0.000].

**Table 3:** Descriptive statistics and correlations between study Variables

Variables	1	2	3	4
1. Sample	–			
2. SCARED	0.65^**^	–		
3. PR	− 0.25^**^	− 0.10^**^	–	
4. SWL	− 0.35^**^	− 0.26^**^	18^**^	–
M	1.49	44.23	3.29	17.70
SD	0.50	18.11	3.06	6.98

*SCARED* screen for child anxiety-related disorders, *SWL* satisfaction with life, *PR* peer relationship; * p < 0.05, ** p < 0.01

**Table 4:** ANOVA results: a comparison between local and refugee samples

Variables	Sum of squares	Degrees of freedom	Mean square	*f*	*p*
1. SCARED	327,992.61	1	327,992.61	1745.852	0.000
2. SWL	14,152.88	1	14,152.88	331.593	0.000

*SCARED* screen for child anxiety-related disorders, *SWL* satisfaction with life

Before testing the hypotheses, we conducted a series of confirmatory factor analyses (CFAs) by utilizing Mplus to examine the validity of our measurement model. As shown in Table 5, the model fit indices of the three-factor model showed an acceptable fit [χ^2^(1275, N = 2356) = 4191.28, χ^2^/df = 3,28, root mean square error approximation (RMSEA) = 0.048, comparative fit index (CFI) = 0.90, Tucker‒Lewis index (TLI) = 0.89, Tucker‒Lewis index (SRMR) = 0.040] and were better than other alternative models examined. The adequate cut-off values for these indices were less than 3 for the χ^2^/df, < 0.08 for the RMSEA and SRMR, and > 0.90 for the CFI and TLI [59, 111]. We, therefore, conclude that these results supported the distinctiveness of the measurements used in this study.

**Table 5:** Fit comparisons of alternative factor models

Hypothesized model	χ^2^	sd	χ^2^/df	CFI	TLI	RMSEA	SRMR
Model A	4191.28	1275	3,28	0.90	0.89	0.048	0.040
Model B	6879.54	1223	5,39	0.81	0.80	0.065	0.048
Model C	7755.51	1224	6,33	0.78	0.77	0.069	0.055

*X:* SCARED*; Y:* Satisfaction with Life*; Z: Peer* Relationship Model A: X, Y, Z; Model B: X + Y, Z; Model C: X + Y + Z; N = 2336

### Mediating results

The indirect effect of peer relationships in determining the mediating role of adolescent anxiety on life satisfaction was examined using Mplus. In this stage, the mediation-moderation model was tested (see Fig. 1). Given that the model is a fully saturated model, the fit indices were not reported. The chi-square statistic for model fit was significant (*p* = 0.000). Additionally, the RMSEA and SRMR values being less than 0.08 within the 95% confidence interval indicate a good model fit.

The analysis results revealed a statistically significant negative relationship between adolescent anxiety and life satisfaction (*B* = − 0.425, *p* = 0.000). Furthermore, a significant relationship was found between peer relationships and adolescent anxiety (*B* = − 0.016, *p* = 0.000). Moreover, our findings demonstrate that the indirect effect of adolescent anxiety on life satisfaction through peer relationships is significant (*B* = 0.194, *p* = 0.000). The confidence interval values [95% CI (0.116–0.273)] did not include zero, indicating a significant indirect effect. The values related to the mediation-moderation model are presented in Table 6.

**Table 6:** Mplus results for the moderated mediation effects

Variables	Standardized estimate	Standardized Error	*p*	Confidence intervals		*f*^2^
				LL	UL	
Variables						
1. SCARED	− 0.425	0.029	0.000	− 0.481	− 0.369	
2. PR	0.194	0.040	0.000	0.116	0.273	
3. Sample	− 16.154	0.987	0.000	− 18.087	14.220	
4. SCARED × Sample	0.270	0.020	0.000	0.231	0.309	0.24

*SCARED* screen for child anxiety-related disorders, *PR* peer relationship, *LL* lower limit with 95% confidence interval; UL: Upper limit with 95% confidence interval; *f*^2^; Cohen's f squared

### Moderation results

**Fig. 2:**
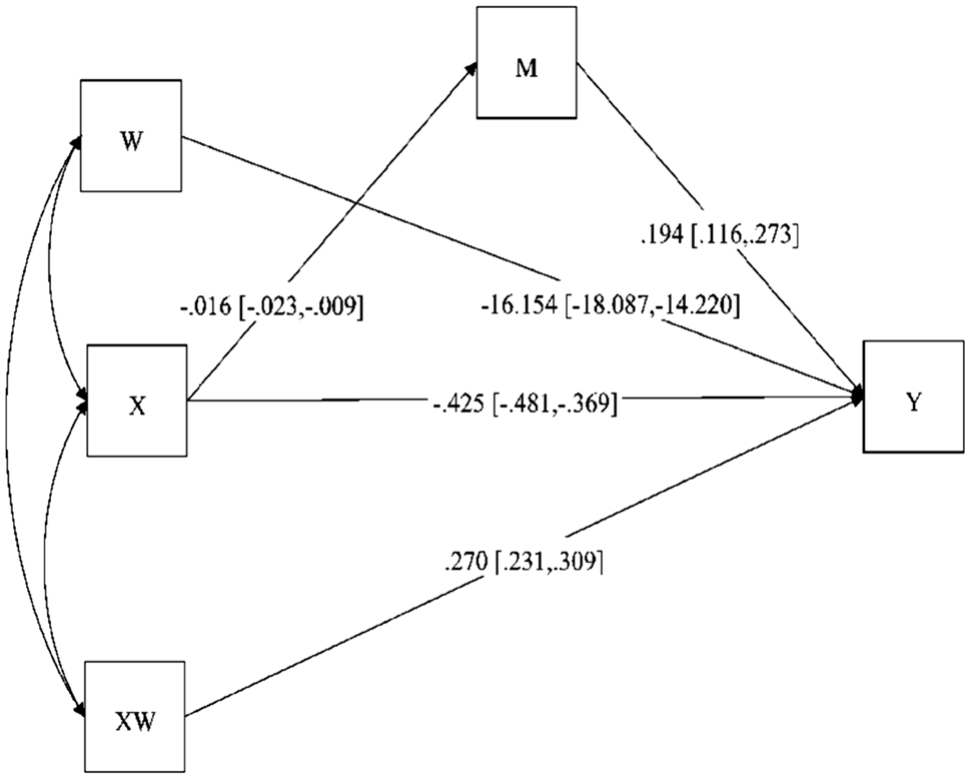
Mplus diagram for moderated mediation. *X* anxiety, *Y* satisfaction with life, *M* peer relationship, *W* participants sample, *XW* anxiety × participant sample interacting term

**Fig. 3:**
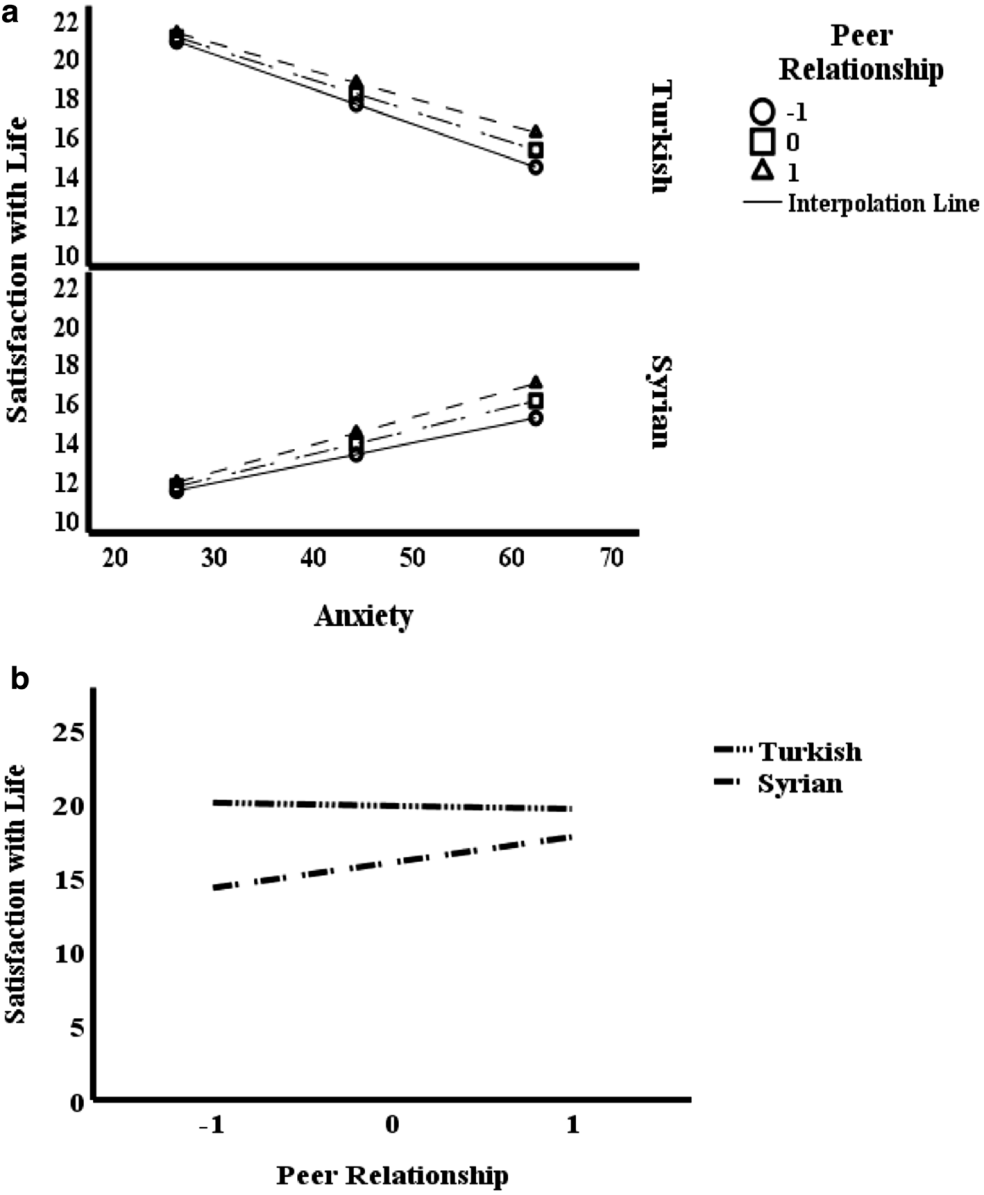
The moderating effect of peer relationship on life satisfaction and anxiety

Lastly, this study examines the moderating role of peer relationships in the indirect effect of adolescent anxiety on life satisfaction within the participant sample. To investigate this relationship, the interaction between adolescent anxiety and the participant sample was initially examined, revealing that this interaction term was positively and significantly related to life satisfaction (*B* = 0.270, p = 0.000). It was concluded that the indirect effect of adolescent anxiety on life satisfaction through peer relationships was determined to a high degree by the participant sample, with an effect size of 0.27 [95% CI (0.231–0.309)]. Additionally, Fig. 2 gives a visual demonstration of the conditional effects of the values (min- and high) of the mediator. The f^2^ statistic was used to calculate the effect size of the interaction term [2]. Accordingly, f^2^ value was calculated as 0.24. This value was interpreted as a moderate effect in line with Cohen's guidelines for interpreting the f^2^ [28]. Following the significance of the moderated mediating effect, we adhered to Cohen et al.'s [29] suggestions in Fig. 3a and b. The visualization illustrates the moderating effect of peer relationships on the relationship between anxiety and life satisfaction among local Turkish and Syrian refugee adolescents. We plotted and interpreted the nature of the two slopes in these multiple models.

## Discussion

This study aimed to investigate the mediating role of peer relationships in the relationship between adolescent anxiety and life satisfaction among Syrian refugee adolescents who sought refuge in Turkey due to the Syrian civil war, which is one of the most significant humanitarian crises in recent history, and local Turkish adolescents. Additionally, the potential moderating effect of being a refugee or a local adolescent on this relationship is investigated. The findings of our study confirm the expected negative relationship between adolescent anxiety and life satisfaction, as well as the negative relationship between adolescent anxiety and peer relationships, which are consistent with relevant literature. Furthermore, our results validate the mediating role of peer relationships in the relationship between adolescent anxiety and life satisfaction.

The current study is in line with previous research conducted on Syrian refugee adolescents who were forced to migrate to Turkey and other countries due to war [64, 68], Llyod 2019, [66, 142]. Consistent with these previous studies, it has been determined that Syrian refugee adolescents exhibit high levels of anxiety symptoms. In addition, consistent with previous studies comparing refugee adolescents and local adolescents in the same region [37], our study found that Syrian refugee adolescents have higher rates of anxiety compared to the local sample [88, 146]. Furthermore, in line with the literature, our findings indicate that the life satisfaction of Syrian refugee adolescents is lower than that of the local sample [3]. Moreover, consistent with the findings of previous studies, a negative relationship between life satisfaction and anxiety symptoms has been identified [7, 63, 129].

The main assumption of this study is that the role of peer relationships in the relationship between adolescent anxiety and life satisfaction will vary depending on whether the participants are native adolescents or refugee adolescents. Adolescence is when young people increasingly distance themselves from their parents, and their relationships with peers become more important [75, 138]. During this period, refugee adolescents, in particular, need social support more than native samples [72]. Refugee adolescents are at a higher risk for psychiatric disorders compared to local samples [134]. Many of the psychiatric disorders experienced during this period tend to be long-lasting and persistent [94]. The negative experiences with peers during this period continue to have an impact in adulthood [112], and they can also serve as precursors to many psychiatric disorders [85, 112].

Studies have shown that a significant portion of refugees lack access to mental health services [76, 102]. However, despite the high rates of psychiatric disorders observed in refugee adolescents, it should not be overlooked that a considerable portion of this population does not develop any mental health problems. This highlights the importance of focusing on the protective factors that preserve the mental health of refugee adolescents [109].

Due to various negative factors that can impact adolescents' adjustment following forced migration, understanding the role of peer relationships is crucial. Literature has consistently highlighted the significance of peer relationships in shaping developmental outcomes during childhood and adolescence [77, 132]. For example, Kan and McHale [65], Victor et al. [132], and Salk et al. [107] suggest that adolescents who do not have healthy peer relationships are more likely to exhibit internalizing problems such as depression and anxiety. Adolescents who are forced to migrate may experience stressors related to relocation [30], acculturation [89], and transitioning to a new school environment [61]. Peer relationships can enhance adolescents' self-esteem and coping skills, while such relationships can also potentially mitigate internalizing problems commonly experienced during adolescence [122, 136]. Following this perspective, problems with peers during adolescence can indicate future problems [60]. This is particularly relevant for vulnerable samples, such as refugees [112]. Support from peers positively affects the post-migration adjustment process for adolescents [9].

It is crucial to identify factors that can promote the adaptation of adolescents who experience stressful life events and difficulties during their migration to a host country [128]. Previous studies have shown the role of different psychological factors in supporting the psychological well-being of Syrian adolescents against stressful life events [143]. Consistent with the available literature, the present study highlights the potential protective effect of peer relationships to alleviate anxiety symptoms and increase life satisfaction among Syrian refugee adolescents.

When examining Fig. 3a, which illustrates the impact of peer relationships on life satisfaction, it can be observed that an increase in peer relationships in the Syrian refugee adolescent sample leads to a more significant increase in life satisfaction than in the local sample. Similarly, when examining Fig. 3b, which visualizes the relationship between life satisfaction and adolescent anxiety based on whether they are local or refugee adolescents, it can be seen that strong peer relationships in Syrian refugee adolescents have a positive effect on increasing life satisfaction in the context of the anxiety-life satisfaction relationship. As a result, this study, in line with the existing literature, highlights the potential protective effect of focusing on peer relationships in alleviating anxiety symptoms and improving low levels of life satisfaction experienced by Syrian refugee adolescents.

According to our findings, one possible reason why peer relationships in local samples do not lead to significant changes in life satisfaction as they do in Syrian refugee adolescents could be attributed to the fact that the local sample benefits from different psychosocial support sources, such as established strong family ties and interactions with peers in different social environments. On the other hand, Syrian adolescents may experience disruptions in their families after resettlement, and their primary sources of socialization may be limited to the school environment and the social interactions within it. This suggests that peer relationships may be more explanatory for this sample.

## Contributions

This study adds to the existing literature by examining the moderator effect of being a native adolescent or refugee adolescent on the relationship between adolescent anxiety and life satisfaction in both native and refugee adolescents residing in the same region. To our knowledge, this study is one of the first to examine this relationship in the context of Syrian refugee adolescents in Turkey. Furthermore, the findings suggest that peer relationships play a crucial role in promoting well-being, specifically life satisfaction, in refugee adolescents. This study sheds light on the importance of peer relationships for refugee adolescents in a host country.

These results have important implications for developing well-being intervention programs for refugee adolescents that target their peer relationships. However, it is important to note that native adolescents may also face peer problems and may require social support, in addition to adaptation problems caused by forced migration. Future research from this perspective could demonstrate the effects of native adolescents’ peer problems on the anxiety-life satisfaction relationship. Overall, the current findings underscore the significance of peer relationships in enhancing the mental well-being of Syrian refugee adolescents living in Turkey. The results support designing interventions that target the development of positive peer relationships to promote mental health among refugee adolescents.

Ecological Systems Theory (EST) is a conceptual model that proposes the importance of considering environmental conditions alongside individual and genetic factors [22]. EST includes four interrelated layers (micro, meso, exo, and macro systems) that must be considered together. Research emphasizes the importance of considering all layers to fully understand immigrant youth's social interactions and experiences [124, 125]. One example is Community-Based Participatory Research (CBPR), in which researchers encourage community-based organizations and stakeholders to participate in the research process to create culturally relevant interventions [11, 34]. Adopting the Community-Based Participatory Research (CBPR) approach in future research may contribute to developing intercultural sensitivity and forming safe peer spaces and social networks with the participation of social stakeholders.

It is emphasized that qualitative research is necessary to understand the relationship between experiences or interactions related to the social environment and psychiatric symptoms during adolescence [78]. Qualitative research involving focus groups includes inductive approaches that reveal the subjective experiences of individuals and the common qualities shared by the group through narrative and discourse analyses [98]. Among these qualitative approaches, Interpretative Phenomenological Analysis (IPA), which originates from a theoretical, philosophical foundation, stands out as a comprehensive practical method that guides the emergence of individuals' processes in making sense of their subjective and social experiences [116, 117].

With the increasing prevalence and importance of online interviews in recent years, the Online Interpretative Phenomenological Analysis (OIPA) method, which is based on IPA in analyzing the data obtained through Online Voice Photo (OPV), is frequently used in studies on youth [36, 119, 126]. Using the OIPA method, researchers aim to achieve effective results by revealing the experiences of youth related to their cultural identities at the micro, meso, exo, and macrosystem levels [127]. Therefore, there is a need for a comprehensive evaluation of the data we have obtained through qualitative studies to be conducted with Syrian refugee adolescents.

## Limitations

This study has some limitations that need to be considered when interpreting the findings. Firstly, since the data collection process was conducted in a school setting, the findings cannot be generalized to adolescents who live in refugee camps, receive education in temporary educational institutions, or do not attend school. Secondly, measures of adolescent anxiety, life satisfaction, and peer relationships were only assessed through self-report measures. Self-report measures may be subject to response bias or social desirability bias. Therefore, the results may not replace direct assessments by mental health professionals, and caution should be exercised in interpreting the results.

Another limitation of this study is the Cronbach’s alpha internal consistency coefficient of our peer problem subscale, which yielded a lower-than-desired value. The fact that the Cronbach’s alpha value of the subscale in the Syrian sample was 0.40 poses a problem regarding scale reliability. However, in the Turkish sample, the Cronbach’s alpha value of the subscale was higher (0.70) than that in other studies. Since this subscale is known to be associated with loneliness and social desirability in adolescents, we obtained significant results in our study despite this limitation.

Lastly, the study design restricts the interpretation of the results regarding causal relationships. Although path analysis and moderation analysis were conducted to examine the relationships among variables and the moderating role of peer relationships, the study's cross-sectional design does not allow us to conclude the direction of the relationships or the causal effects of the variables on each other. Therefore, future studies should use longitudinal designs to establish temporal relationships among variables and examine causal relationships.

## Data Availability

Not applicable.
